# Visible-near infrared hyperspectral imaging for non-destructive estimation of leaf nitrogen content under water-saving irrigation in protected tomato cultivation

**DOI:** 10.3389/fpls.2025.1676457

**Published:** 2025-10-01

**Authors:** Caixia Hu, Tingting Zhao, Yingying Duan, Yungui Zhang, Xinxiu Wang, Jie Li, Guilong Zhang

**Affiliations:** ^1^ Agro-Environmental Protection Institute, Ministry of Agriculture and Rural Affairs, Tianjin, China; ^2^ Institute of Agricultural Resources and Regional Planning, Chinese Academy of Agricultural Sciences, Beijing, China

**Keywords:** tomato, leaf nitrogen content (LNC), hyperspectral imaging, water-saving irrigation, machine learning

## Abstract

Accurate estimation of leaf nitrogen content (LNC) is critical for optimizing fertilization strategies in greenhouse tomato production. This study developed a robust hyperspectral-based framework for non-destructive LNC prediction by combining advanced spectral preprocessing, feature selection, and machine learning. Hyperspectral reflectance data were collected across five nitrogen and irrigation treatments over key growth stages. Signal quality was enhanced through Savitzky–Golay smoothing (SG) and Standard Normal Variate normalization (SNV). Key nitrogen-sensitive wavelengths—centered around 725 nm and 730 - 780 nm—were identified using Competitive Adaptive Reweighted Sampling (CARS) and Principal Component Analysis (PCA). Four predictive models were compared, among which a hybrid Stacked Autoencoder–Feedforward Neural Network (SAE-FNN) achieved the highest accuracy (test R² = 0.77, RPD = 2.06), effectively capturing nonlinear spectral–nitrogen interactions. In contrast, Support Vector Machine (SVM) exhibited overfitting and Partial Least Squares Method (PLSR) underperformed due to its linear constraints. These results underscore the potential of integrating hyperspectral sensing with deep learning for intelligent nitrogen monitoring in controlled-environment agriculture.

## 1 Introduction

Tomato (*Solanum lycopersicum* L.) is a globally cultivated vegetable crop, valued for its nutritional and economic importance (FAO, 2023). Among the various agronomic inputs, nitrogen plays an essential role in plant growth and development, as it is a core component of amino acids, proteins, chlorophyll, and nucleic acids (Leitner, 2011; Zhu and Chen, 2002). However, the interaction between N availability and irrigation, particularly under water-saving practices, complicates nutrient uptake dynamics. Traditional N management strategies, which rely on field experience and soil testing, often fall short in meeting real-time nutrient demands (Cerasola et al., 2025; Xu et al., 2021; Zhai et al., 2022). Therefore, developing non-destructive and phenology-aware approaches to monitor LNC is essential for precise N management in protected tomato production (Song et al., 2016; Zhou et al., 2025).

To address these limitations, visible-near infrared (VIS-NIR, 400–1000 nm) hyperspectral imaging (HSI) has emerged as a powerful tool for non-destructive, growth-stage-adaptive monitoring of crop nutritional status. Compared to conventional multispectral imaging or fluorescence-based techniques, HSI captures both spectral and spatial information, enabling the detection of nitrogen-induced physiological signals at fine scales, such as 550 nm (chlorophyll-a), 680 nm (protein absorption), and 730 nm (red-edge shift) (Pandey et al., 2017; Thenkabail et al., 2000). Prior studies have demonstrated the effectiveness of HSI-based nitrogen monitoring in crops such as tomato, wheat, and rice (Chen et al., 2025; Yu et al., 2020; Zhou et al., 2025).

However, a major limitation of current HSI-based nitrogen estimation models is their reliance on static spectral features derived from single time points or fixed growth stages. These models frequently overlook the temporal dynamics of spectral-physiological relationships that occur during plant development (Liu et al., 2024). Key leaf traits such as chlorophyll content, leaf structure, and water status change significantly throughout growth, leading to stage-dependent shifts in nitrogen-sensitive spectral regions, particularly in the 750–900 nm range (Elvanidi et al., 2018). Additionally, nitrogen remobilization during fruit development alters spectral signatures such as red-edge position. Static models built on fixed spectral bands often exhibit poor generalization across stages and treatments, limiting their operational utility (Way et al., 2017). Moreover, greenhouse environments introduce additional complexities such as heterogeneous lighting, surface water films, and near-infrared stray light, all of which challenge model stability (Katsoulas et al., 2016). Addressing these challenges requires robust experimental controls, in parallel with the development of phenology-aware modeling frameworks that can dynamically adapt to growth stage-dependent spectral variability.

Previous studies have explored various spectroscopic approaches for estimating tomato LNC. Conventional linear multivariate regression methods, such as PLSR and principal component regression, have been widely applied due to their interpretability and effectiveness with small datasets (Asefa et al., 2005; Osco et al., 2019; Song et al., 2011).These studies have successfully identified key nitrogen-sensitive wavelengths, typically found in the visible and red-edge regions. To capture nonlinear relationships, machine learning techniques such as SVM and Random Forest (RF) have been adopted, achieving improved accuracy than linear models (e.g. Ihuoma and Madramootoo, 2020). More recently, deep learning architectures such as convolutional neural networks (CNNs) have demonstrated superior performance in extracting complex features from hyperspectral data for nitrogen assessment in crops like rice and maize (Qiu et al., 2021; Zhang et al., 2023b). However, when applied specifically to tomatoes, several critical gaps remain. Firstly, many models are calibrated at a single or limited growth stage, overlooking the physiological and spectral changes that occur throughout the tomato lifecycle (Liu et al., 2024). Secondly, few models consider the combined effects of water and nitrogen stress, which are common in water-saving greenhouse cultivation (Rubo and Zinkernagel, 2022). Consequently, a robust, stage-adaptive framework is needed to capture spectral dynamics and disentangle water–nitrogen interactions in tomato canopies.

This study integrates hyperspectral imaging with machine learning to develop a phenology-aware framework for tomato nitrogen monitoring. This study focuses on capturing dynamic nitrogen-related spectral responses across growth stages, aiming to provide real-time insights into nitrogen status that support more informed and efficient fertilization strategies. We hypothesize that dynamic selection of growth-stage-specific sensitive bands, combined with advanced feature extraction techniques, can enhance the robustness of growth status and nitrogen prediction models across critical developmental phases. The objectives of this study are: (1) to identify phenology-dependent spectral features correlated with growth status and nitrogen dynamics in tomato plants. (2) to optimize machine learning models (PLSR, SVM, etc.) for improved accuracy and generalizability under greenhouse vegetable conditions. (3) to validate the practical utility of hyperspectral mapping for precision nitrogen management in greenhouse cultivation. This research is expected to advance the state of remote sensing-based crop nutrient monitoring by providing a robust and phenology-aware modeling framework that supports intelligent decision-making in sustainable protected agriculture.

## 2 Materials and methods

### 2.1 Site description

The experiment was conducted in a solar greenhouse located in Chaomicun Village, Daliang Town, Wuqing District, Tianjin, China (117°2’46″E, 39°32’5″N). The region has a warm temperate monsoon climate, with an average annual precipitation of 532 mm, a mean temperature of 13.5 °C, and an annual sunshine duration of approximately 2,400 hours. The soil was classified as loamy fluvo-aquic with good drainage and irrigation conditions (**Table 1**).

**Table 1:** Basic physicochemical properties of the 0–20 cm plow layer soil.

pH	Electrical conductivity (ds m^-1^)	Soil organic carbon (g kg^-1^)	Total N (g kg^-1^)	NH_4_^+^-N (mg kg^-1^)	NO_3_^–^N (mg kg^-1^)
8.1	0.2	9.6	1.1	29.0	150.4

The tested tomato cultivar was “Provence” (large-fruited type). Tomato seeds were sown in mid-September for seedling production and subsequently transplanted to the greenhouse in early November. The fruiting period was managed using a standard single-stem pruning system, with the main stem trained to retain seven fruit trusses (inflorescences). Harvesting of the first truss commenced in late February, followed by sequential harvesting of subsequent trusses. The second and third trusses were harvested from mid-March to mid-April. The crop was terminated after harvesting the seventh truss in mid-May.

### 2.2 Experimental design

The experimental design included three fertilization levels: unfertilized control, optimized fertilization, and conventional fertilization. Among them, the optimized fertilization applied 65% of the nitrogen used in conventional fertilization, which was based on the average nitrogen application rates typically used in local facility vegetable fields (**Table 2**).

**Table 2:** Nitrogen application rates for conventional and recommended fertilization practices.

Fertilizer	Month	Organic fertilizer N (kg ha^-1^)	Chemical fertilizer N (kg ha^-1^)	Total N input (kg ha^-1^)
Recommended fertilization	November	450	0	450
	December	0	0	0
	January	0	32.59	32.59
	February	0	97.78	97.78
	March	0	97.78	97.78
	April	0	43.46	43.46
Total		450	271.61	721.61
Conventional fertilization	November	787.50	0	787.50
	December	0	0	0
	January	0	55.56	55.56
	February	0	166.67	166.67
	March	0	166.67	166.67
	April	0	74.07	74.07
Total		52.50	462.97	1250.47

Three irrigation regimes were implemented: full irrigation (100% of irrigation water demand, IWD), 25% water-saving irrigation (75% IWD), and 50% water-saving irrigation (50% IWD). Water-saving irrigation treatments were applied only in combination with optimized fertilization (**Table 3**).

**Table 3:** Monthly irrigation requirements for tomato growth under different irrigation depths (100%, 75%, and 50% of IWD).

Month	100% IWD (m^3^ ha^-1^)	75% IWD (m^3^ ha^-1^)	50% IWD (m^3^ ha^-1^)
November	0	0	0
December	270	202.5	135
January	180	135	90
February	540	405	270
March	450	337.5	225
April	180	135	90
Total	1620	1215	810

The experimental treatments were as follows: T1: No fertilization + 100% IWD; T2: Conventional fertilization + 100% IWD; T3: Optimized fertilization + 100% IWD; T4: Optimized fertilization + 75% IWD; T5: Optimized fertilization + 50% IWD.

The base fertilizer only used organic fertilizer (a mixture of chicken manure and cow manure in a 1:2 ratio), with nitrogen and phosphorus contents of 1.5% and 1.6%, respectively. Topdressing was using a water-soluble fertilizer with a nitrogen, phosphorus, and potassium (N-P_2_O_5_-K_2_O) ratio of 15-15-10. Topdressing was applied every 5–7 days during the peak growth period of tomato. The experiment was conducted in a randomized complete block design with three replicates per treatment. Each plot measured 9 m × 5 m (3 planting ditches, 6 crop rows), with 1 m spacing between plots. The nitrogen levels and irrigation treatment were designed based on local practices and previous studies, in order to investigate the interactive effects of these factors on nitrogen uptake and utilization efficiency in tomatoes (Gong et al., 2022).

### 2.3 Sample collection and laboratory analysis

**Figure 1:**
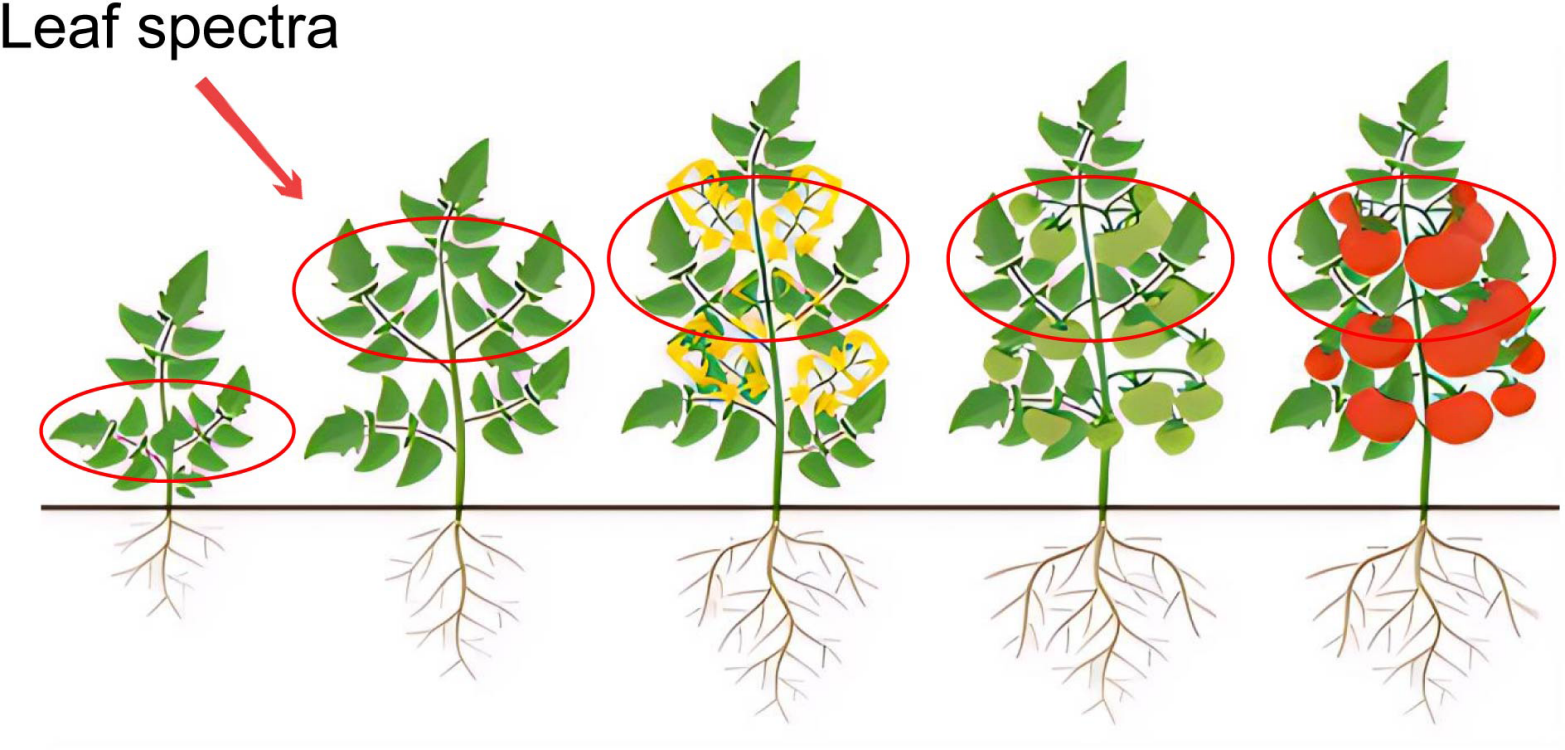
Schematic illustration of Regions of Interest (ROIs) selected on functional leaves of tomato plants at different growth stages. The circled areas indicate the leaf positions used for hyperspectral data acquisition to represent nitrogen status.

Tomato samples were collected five times during key growth period, including seedling, flowering, fruit setting, swelling, and maturity stage. For each treatment, three plants were randomly selected, and the entire plant was destructively sampled. Regions of Interest (ROIs) for hyperspectral measurements were chosen from functional leaves located in the upper–middle canopy, while avoiding senescent or damaged leaves (**Figure 1**). At each sampling event, 2–3 such leaves were used for spectral acquisition.

Hyperspectral reflectance (400–1000 nm) was measured using a SHIS - N220 (Shenzhen Zhongdarui Technology Co., Ltd., China) hyperspectral imaging system, and SPAD values (as a proxy for chlorophyll content) were recorded using a SPAD - 502 (Konica Minolta, Inc., Japan.) chlorophyll meter (Konica Minolta). Following spectral measurements, leaf was oven-dried at 105 °C for 30 minutes, then dried at 65 °C until constant weight was achieved. The dried samples were subsequently analyzed for total nitrogen content. A 0.2 g subsample was weighed and detected using the H_2_SO_4_ - H_2_O_2_ method until the solution became clear. The digest was diluted to 50 mL, and total nitrogen content (%) was determined using a Kjeltec nitrogen analyzer (FOSS Analytical A/S, Denmark.) (Pieters et al., 2020).

### 2.4 Hyperspectral image acquisition and processing

Hyperspectral data were acquired using a push-broom visible near-infrared (VNIR) hyperspectral imager (Model: SHIS - N220 (Shenzhen Zhongdarui Technology Co., Ltd., China), spectral range: 400–1000 nm, spectral resolution: 3 nm, 200 spectral bands). The system was equipped with a full-spectrum halogen lamp (effective wavelength range: 400–1000 nm), optically filtered to match the instrument’s operational band (400–1000 nm), and housed within a dark enclosure to minimize ambient light interference.

Calibration Protocol: 1) Dark Current Calibration: A black reference image (0% reflectance) was acquired to correct for sensor noise and dark current. 2) White Reference Calibration: A high-reflectivity whiteboard (Spectralon^®^, >99% reflectance) was imaged to standardize reflectance values across the spectral range. 3) Reflectance Calculation: Raw hyperspectral data were normalized using the following equation (Equation 1):


(1) $$I(i,\;j)=\frac{R_{raw}(i,\;j)-R_{black}(i,\;j)}{R_{white}(i,\;j)-R_{black}(i,\;j)}$$

$R_{raw}$ , $R_{black}$ , $R_{white}$ are the original image, blackboard, and whiteboard reflection values, respectively.

During each sampling event, ROIs were manually delineated on the functional leaves to extract average spectral reflectance values.

ROIs corresponding to the leaf area were extracted using adaptive threshold segmentation. The average spectrum of the leaf region was subsequently computed. To reduce spectral noise and improve signal quality, the Savitzky-Golay smoothing filter (SG), first-order derivative, and Standard Normal Variate (SNV) transformation were applied as preprocessing steps (Raj et al., 2021).

### 2.5 Feature selection and dimensionality reduction

To address spectral redundancy and enhance model robustness, two complementary methods, PCA, and CARS—were applied for feature extraction and dimensionality reduction. PCA transformed the high-dimensional spectral data into a set of orthogonal principal components (PCs), emphasizing wavelengths contributing most to overall variance. CARS iteratively selected wavelengths by weighting PLS regression coefficients and applying Monte Carlo sampling to adaptively optimize band selection (Li et al., 2009).

### 2.6 Machine learning modeling

Four machine learning models were implemented and compared to predict leaf nitrogen content based on hyperspectral data.

#### 2.6.1 Partial least squares method

PLSR effectively handled multicollinearity among spectral variables by identifying latent components that maximized the covariance between the spectral data (X) and the nitrogen concentration (Y) (Song et al., 2011). This enabled the model to establish a robust linear relationship for predicting plant nitrogen status.

#### 2.6.2 Support vector machine

SVM **was used** to model non-linear relationships by constructing an optimal hyperplane in a high-dimensional feature space (Asefa et al., 2005). By using suitable kernel functions and optimizing the hyperparameters, it achieves excellent regression performance when predicting nitrogen content based on spectral inputs.

#### 2.6.3 Feedforward neural network

The FNN captured complex nonlinear patterns in hyperspectral data via multiple layers of nonlinear transformation. With structures optimized via experimental or algorithmic tuning and regularized training techniques such as dropout and L_2_ regularization, it maintains a high level of generalization when modelling spectral-biochemical relationships (Wang et al., 2024).

#### 2.6.4 Stacked autoencoder-based feedforward neural network

SAE-FNN utilized unsupervised pre-training to learn informative, low-dimensional representations of spectral data, which improved model initialization and feature extraction (Tao et al., 2015). By emphasizing salient spectral regions (e.g. the red-edge and NIR plateau) and suppressing noise through layered dimensionality reduction and explicit regularization, it enhanced the model’s ability to discriminate between nitrogen levels (Yu et al., 2018).

### 2.7 Model evaluation

The performance of the predictive models was evaluated using commonly adopted metrics in spectral modeling and machine learning, including the coefficient of determination (R²), root mean square error (RMSE), relative percent deviation (RPD), and signal-to-noise ratio (SNR) (Pandey et al., 2017; Raj et al., 2021; Walpole, 1993).

## 3 Results

### 3.1 Analysis of LNC in tomatoes

**Figure 2:**
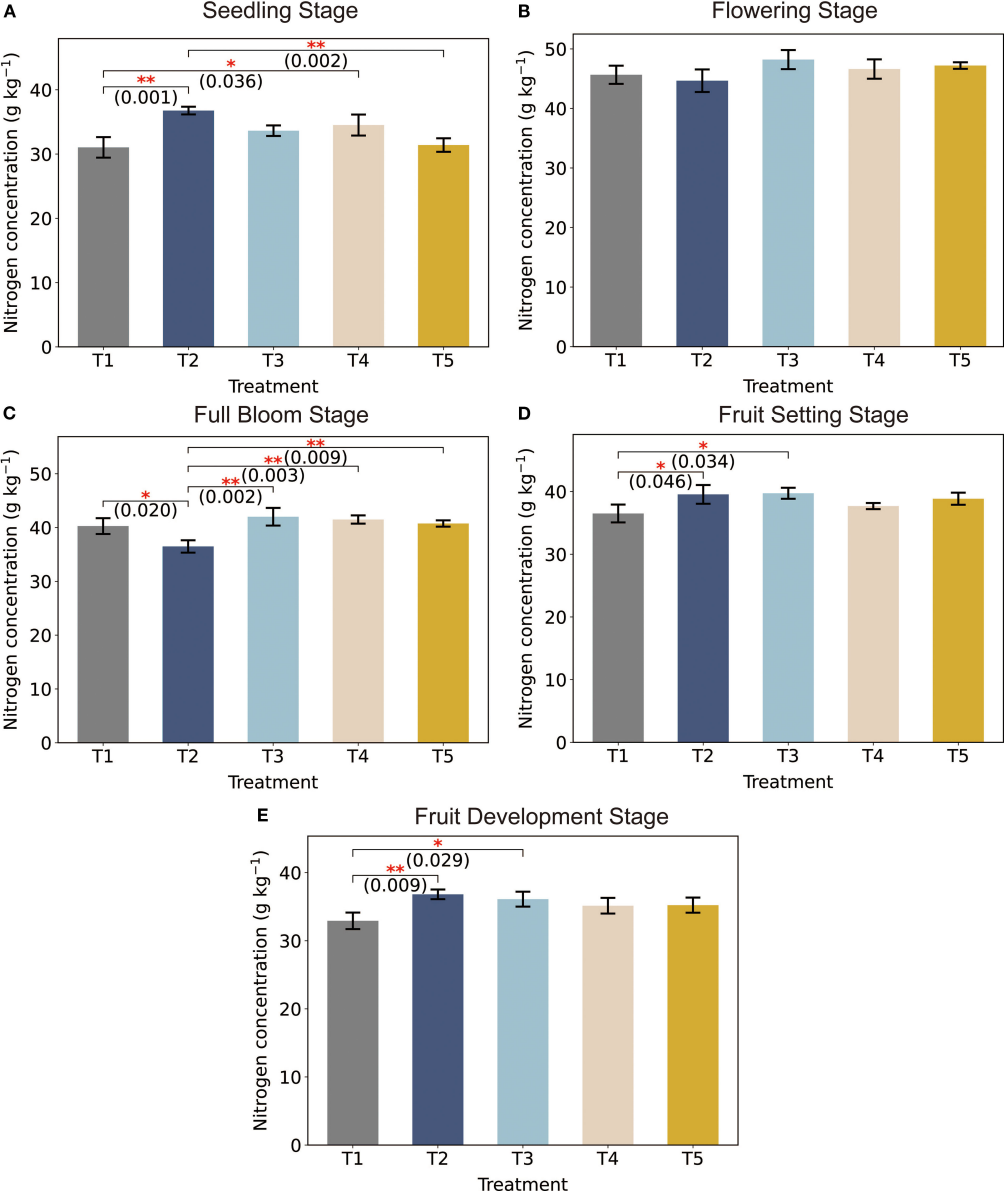
LNC of tomatoes plants under different treatments across developmental stages. Asterisks indicate significant differences between treatments: *p < 0.05, **p < 0.01. The p-values are displayed above the bars to indicate the significance level for each pairwise comparison.

Nitrogen concentration varied significantly among treatments at different growth stages (*p* < 0.05), with values expressed as mean ± standard error (**Figure 2**). At the seedling stage (**Figure 2A**), T2 showed the highest nitrogen concentration, which was significantly greater than that of T1 (*p* = 0.001) and T5 (*p* = 0.036), with T5 demonstrating the lowest value. At full bloom (**Figure 2C**), T3 presented the highest nitrogen concentration, significantly exceeding T1 (*p* = 0.020) and T2 (*p* = 0.009), whereas T4 and T5 did not differ significantly (*p* > 0.05).

During fruit-setting stage, although T1 had the highest nitrogen concentration (**Figure 2D**), the differences among treatments were not statistically significant (*p* > 0.05). In the fruit development stage, T2 again showed the highest nitrogen concentration (**Figure 2E**), significantly greater than that of T1 (*p* = 0.009) and T5 (*p* = 0.029), while T5 consistently exhibited the lowest nitrogen levels. These results indicate that plant nitrogen accumulation was not only treatment-dependent but also strongly stage-specific. The temporal shifts in peak nitrogen concentrations across treatments suggest that different management strategies may influence nitrogen uptake efficiency at distinct phenological stages.

SPAD values, which serve as a proxy for relative chlorophyll content, closely mirrored the variations in LNC observed at different stages of treatment and growth (**Supplementary Figure S1**). T2 exhibited the highest SPAD values throughout all developmental stages. Conversely, T1 exhibited the lowest readings. A progressive decline in SPAD values was evident from the seeding stage to the development stage across most treatments. Maximum values generally occurred during the flowering stage, followed by a gradual reduction as the plants progressed through the flowering, full bloom and fruit-setting stages.

The tomato leaf samples were sorted in ascending order based on nitrogen content and then partitioned into training and testing sets at an 80:20 ratio, resulting in 60 training samples and 15 testing samples. Descriptive statistics for the dataset are presented in **Table 4**. The nitrogen content across all 75 tomato leaf samples ranged from 30.79 to 49.49 g kg^-1^, with an average of 38.74 g kg^-1^ and a standard deviation of 4.96 g kg^-1^, indicating moderate variability. The nitrogen concentration range in the training set was 39.21 to 49.49 g kg^-1^, while that in the testing set was 30.14 - 46.95 g kg^-1^. The dataset exhibited a reasonable distribution and moderate variability, meeting the requirements for model development. Therefore, the spectral characteristics of tomato leaves under different treatments and growth stages were further analyzed to explore their relationships with nitrogen content.

**Table 4:** Descriptive statistics of tomatoes LNC (g kg^-1^).

Statistic	Count	Mean	Std	Min	Q1	Median	Q3	Max
Total sample	75	38.74	4.96	30.09	35.35	37.76	41.47	49.49
Train set	60	39.21	4.94	30.09	35.55	38.83	41.81	49.49
Test set	15	36.85	4.70	30.14	33.86	36.37	37.80	46.95

Std: Standard deviation; Min: Minimum; Q1: 25th percentile; Q3: 75th percentile; Max: Maximum.

### 3.2 Analysis of hyperspectral reflectance data

**Figure 3:**
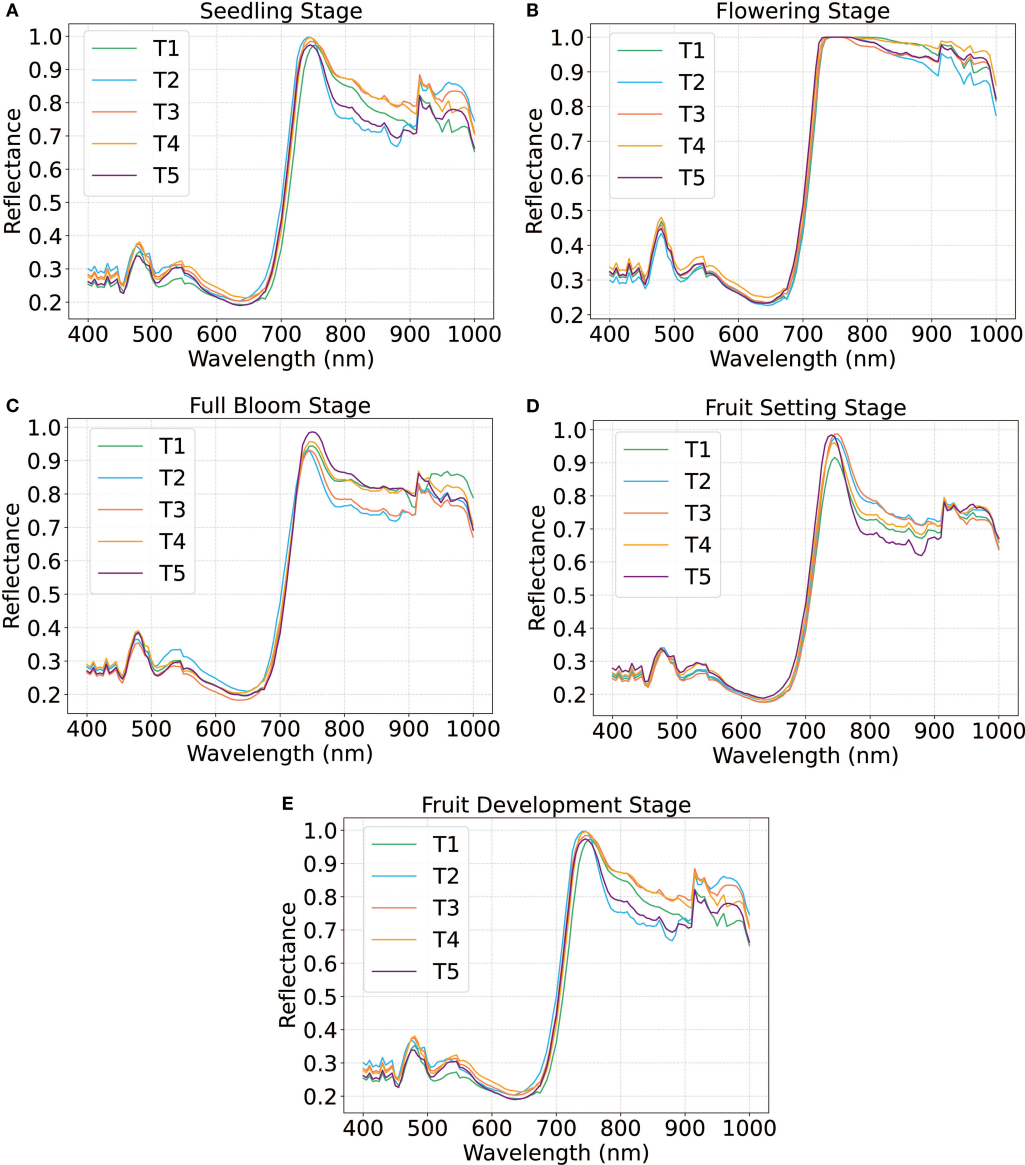
Spectral response of the leaf with different treatment at different periods.

**Figure 3** illustrated the hyperspectral reflectance spectra of tomato plants at various growth stages under five treatments (T1-T5), with reflectance plotted as a function of wavelength (nm). Each line represents the spectral response of plants under a specific treatment.

The reflectance spectra revealed both stage-dependent and treatment-specific variations that closely linked to physiological dynamics throughout tomato plant development. At the seedling stage, spectral patterns were largely similar across treatments, with a prominent reflectance peak near 700 nm, where T2 exhibited marginally higher reflectance (**Figure 3A**).

During the flowering stage (**Figure 3B**), reflectance increased across all treatments, with T3 displaying the highest reflectance across most wavelengths, while T4 and T5 exhibited an obvious decline in reflectance above 700 nm. By full bloom, spectral divergence among treatments became more pronounced (**Figure 3C**). T3 displayed maximal reflectance in the 600–700 nm, corresponding to the chlorophyll absorption band, indicating a higher pigment content or delayed senescence. contrasting However, T5 showed minimal reflectance and T1 and T2 had the intermediate values.

In the fruit setting stage (**Figure 3D**), overall reflectance declined, but T1 displayed the highest spectral values. This trend shifted again during fruit development (**Figure 3E**), with T2 emerging as the dominant treatment, while T4 and T5 consistently exhibited the lowest reflectance across both stages. These dynamic spectral variations underscore the impact of treatment on plant physiological status across growth phases. The sensitivity of hyperspectral reflectance, particularly in the red-edge and chlorophyll absorption regions, suggesting its value as a non-destructive tool for real-time monitoring of crop status under varying management regimes.

**Figure 4:**
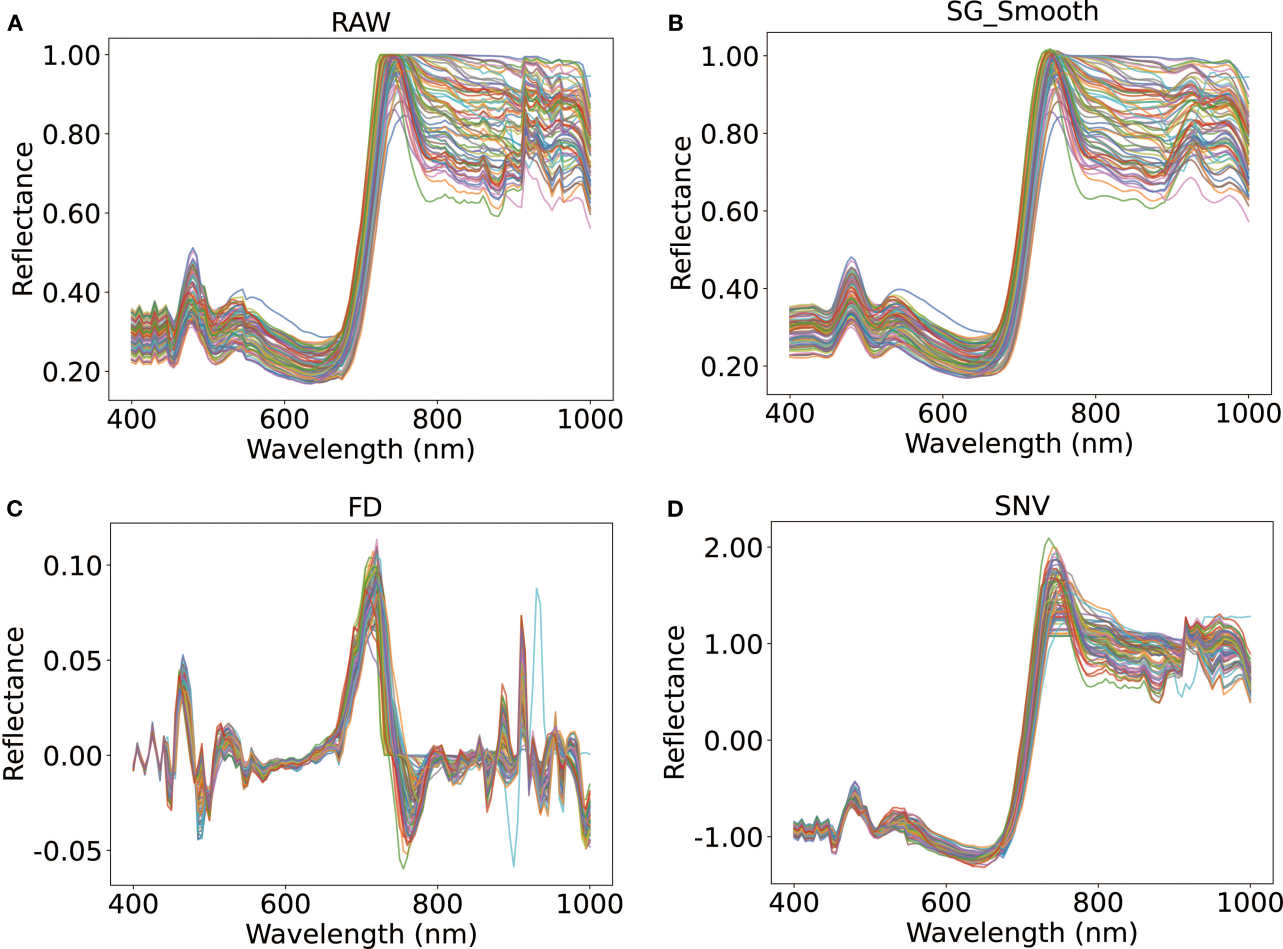
Comparison of pretreatment methods: **(A)** Raw; **(B)** Savitzky-Golay smoothing filter (SG_Smooth); **(C)** First-Derivative (FD); **(D)** Standard Normal Variate (SNV).

The hyperspectral reflectance data of tomato leaves were processed using multiple preprocessing techniques to enhance spectral quality and prepare the data for robust modeling (**Figure 4**). The raw reflectance curves (**Figure 4A**) exhibited inherent variability and minor outliers, which could compromise signal integrity and model performance. Therefore, preprocessing was necessary to suppress noise and standardize the data. SG effectively reduced high-frequency noise, resulting in smoother spectral curves while retaining key spectral features (**Figure 4B**).

Subsequently, the first-derivative transformation (**Figure 4C**) was applied to accentuate wavelength-dependent slope variations, revealing subtle variations in spectral shape that were not apparent in raw data. To further correct for baseline offsets and scale differences, SNV normalization (**Figure 4D**) was employed to center the reflectance values around zero and mitigated baseline shifts, thereby improving sensitivity to nitrogen-related spectral responses.

**Figure 5:**
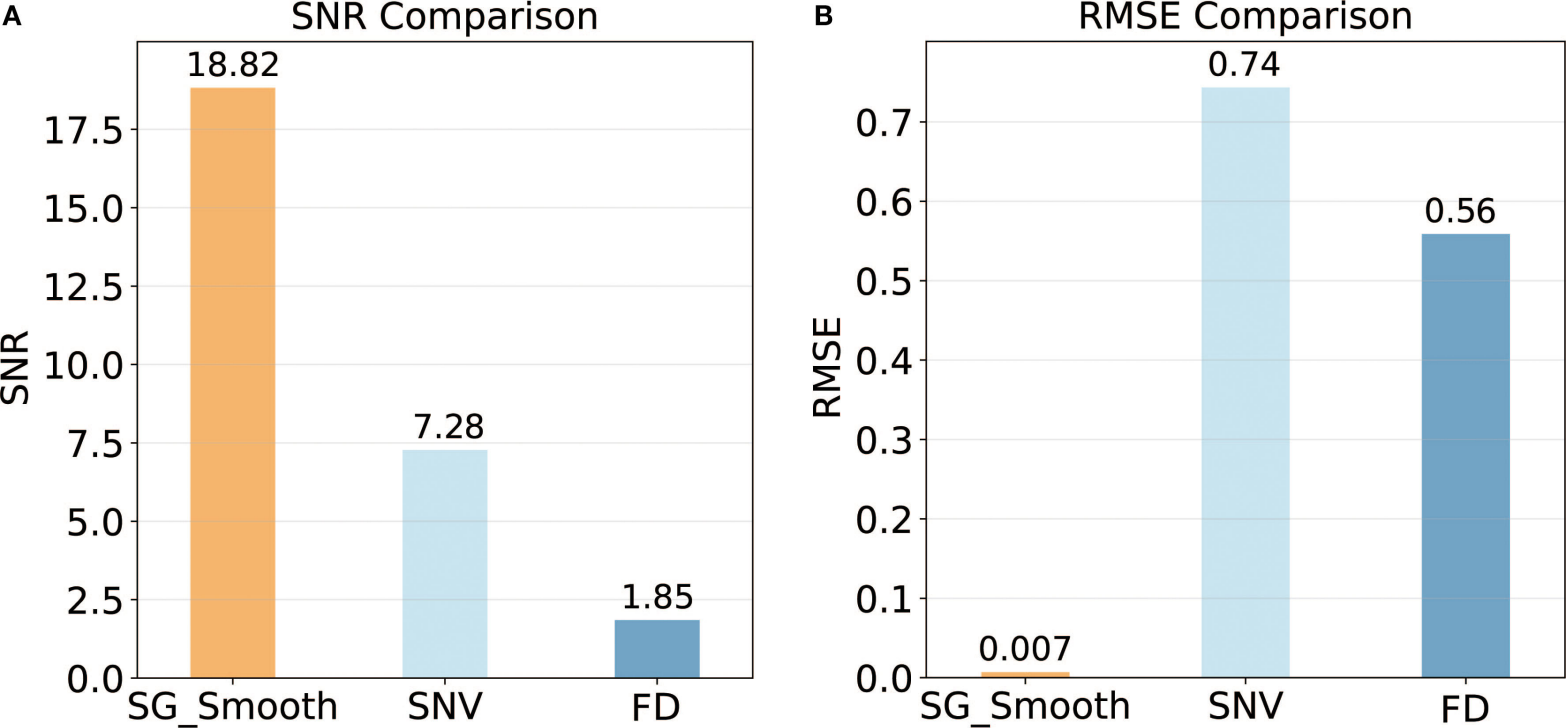
Quantitative comparison of spectral preprocessing methods: **(A)** Signal-to-Noise Ratio (SNR); **(B)** Root Mean Square Error (RMSE).

In addition to the qualitative interpretation, quantitative comparisons confirmed that SG smoothing achieved the highest noise suppression (SNR = 18.824) and the optimal modeling accuracy (RMSE = 0.007) (**Figures 5A, B**). Overall, the application of SG smoothing provided an optimal balance for noise reduction with feature retention, providing robust and consistent spectral inputs for subsequent modeling.

### 3.3 Feature extraction analysis

To improve model efficiency and reduce spectral redundancy, two feature extraction methods—PCA and CARS—were applied to identify the most informative wavelengths associated with LNC. To ensure a single, growth-robust nitrogen-prediction model, spectral preprocessing (SG, FD, SNV) and feature selection (PCA, CARS) were performed once on the full multi-stage dataset.

**Figure 6:**
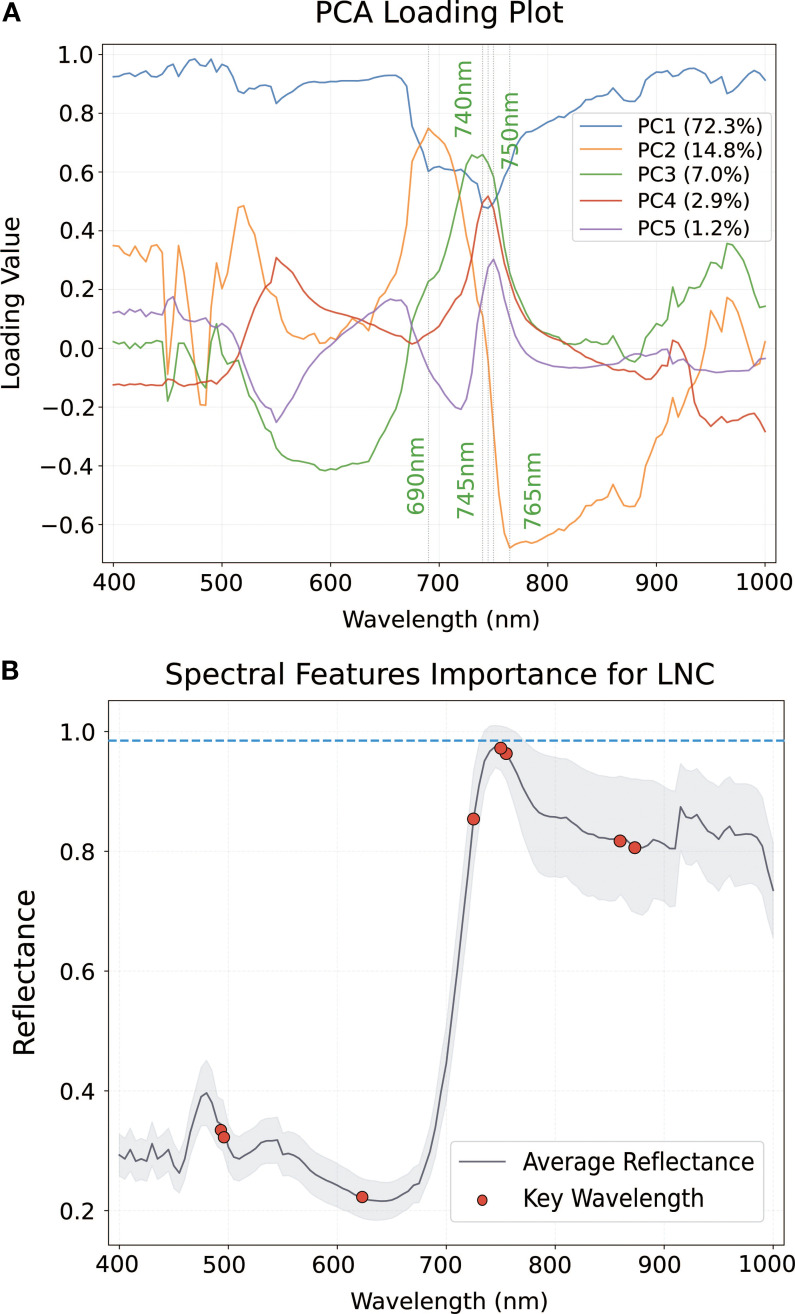
Illustration of results of PCA and CARS: **(A)** Loading weights of wavelengths using the PCA method. Sensitive wavelengths of LNC **(B)** extracted by using the CARS method.

As shown in **Figure 6A**, the PCA loading plot illustrated the contribution weights of spectral wavelengths to the principal components (PCs). The first principal component (PC1), accounting for 72.3% of the total spectral variance, exhibited prominent loading values at 690 nm, 740 nm, 745 nm, 750 nm, and 765 nm (**Figure 6A**). These wavelengths are strongly associated with nitrogen-sensitive spectral regions, particularly in the red-edge and near-infrared domains, indicating their relevance in capturing LNC variability. The subsequent principal components (PC2–PC5) contributed 22.1%, 3.2%, 1.5%, and 0.9%, respectively, demonstrating diminishing explanatory power with increasing component order.

In contrast, the competitive adaptive reweighted sampling (CARS) method was employed to identify predictive spectral variables for LNC (**Figure 6B**). The variable importance plot revealed a distinct peak near 730 nm, corresponding to a region closely aligned with chlorophyll absorption and nitrogen-associated biochemical features. Additional key wavelengths were identified between 730 nm and 780 nm, overlapping with the red-edge spectral region. The shaded background in **Figure 4B** represents mean reflectance spectrum across samples, while red markers indicate wavelengths with the highest variable importance scores (VIS > 0.85), emphasizing localized spectral importance rather than broad variance.

The comparative performance of the two feature extraction methods for LNC prediction revealed significant disparities in model performance (**Table 5**). CARS outperformed PCA, achieving the highest validation accuracy (R_V_^2^ = 0.79, RMSE_V_ = 2.26, RPD_V_ = 2.20), indicating more stable and effective wavelength selection.

**Table 5:** Performance comparison of feature extraction methods (PCA and CARS) evaluated.

Pigment	Method	Np	R_C_^2^	R_V_^2^	RMSE_C_	RMSE_V_	RPD_C_	RPD_V_
LNC	PCA	4	0.36	0.37	3.90	3.93	1.25	1.26
	CARS	8	0.72	0.79	2.60	2.26	1.87	2.20

Np represents the number of principals, i.e., features, used in prediction and the R_C_^2^, R_V_^2^, RMSE_C_, RMSE_V_, RPD_C_, and RPD_V_ denotes the values of R^2^, RMSE, and RPD on train set and test set, respectively.

In contrast, PCA got the intermediate results (R_V_^2^ = 0.37, RMSE_V_ = 3.93), likely due to its limited capacity to capture nonlinear or biochemically localized variations in spectral data. These findings suggested CARS maintained model parsimony with only 8 selected predictors (Np = 8), striking a balance between dimensionality reduction and predictive accuracy.

### 3.4 Performance analysis of models

**Figure 7:**
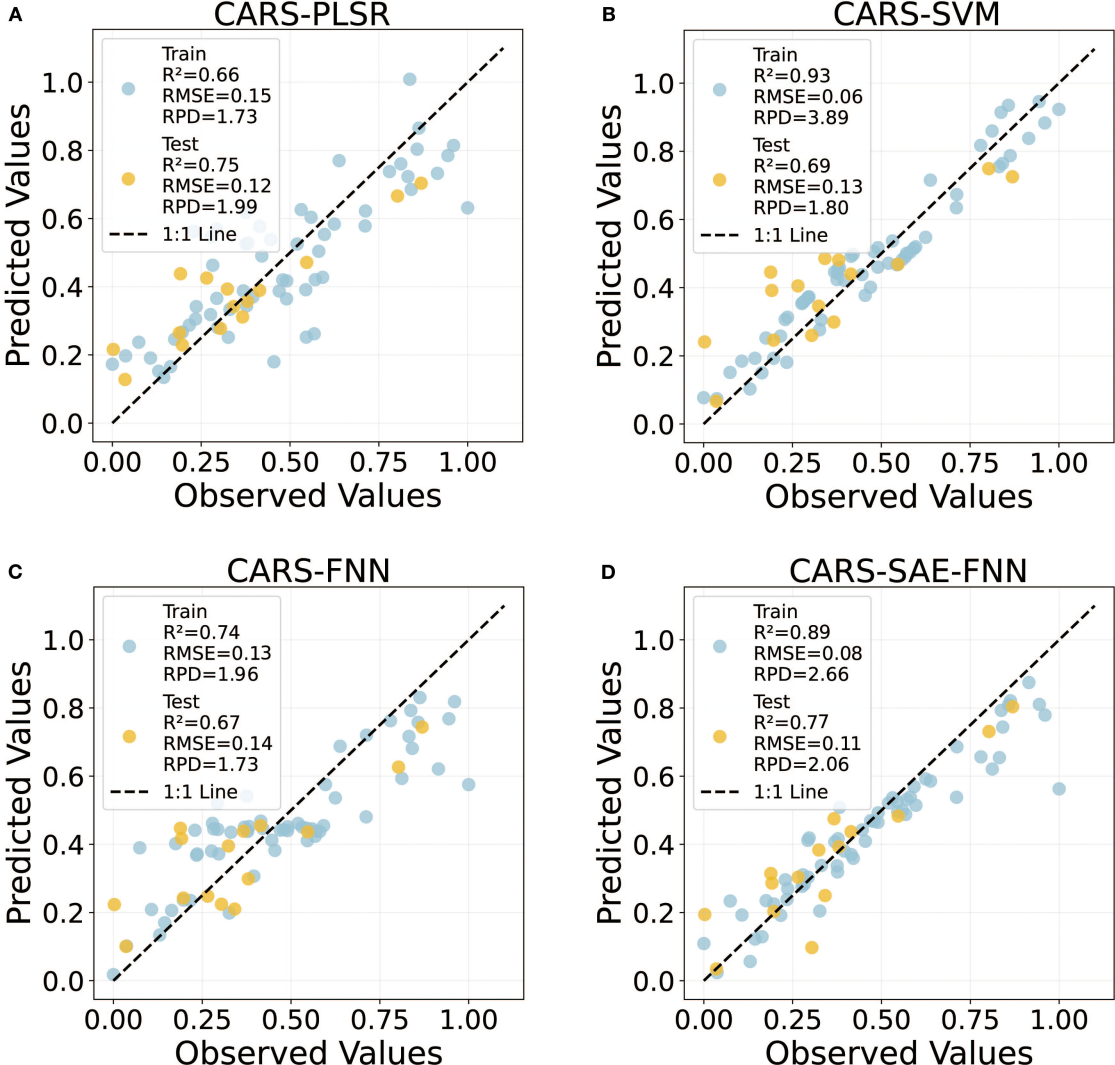
The predictive performance of the four machine learning models (PLSR, SVM, FNN and SAE-FNN) were based on the optimal CARS feature extraction method.

To evaluate the predictive capacity of different algorithms for estimating tomato LNC, four models—SAE-FNN, SVM, PLSR, and FNN—were compared in terms of accuracy, generalization ability, and robustness using both training and testing datasets (**Figure 7**). All models were implemented with their hyperparameters rigorously optimized to ensure a fair comparison (**Supplementary Table S2**).

Among the tested models, the SAE-FNN hybrid demonstrated superior performance, achieving the highest test accuracy (R^2^ = 0.77, RMSE = 0.11, RPD = 2.06) with the minimal divergence between training and testing results (ΔR^2^ = 0.12), indicating good generalization capability (**Figure 7D**). The superior performance of SAE-FNN is attributed to its ability to capture nonlinear and hierarchical spectral patterns via deep stacked autoencoder layers, enabling more effective abstraction of nitrogen-sensitive features from high-dimensional hyperspectral data.

In contrast, the SVM model exhibited significant overfitting, with a sharp decline in test performance (R^2^ = 0.69, RPD = 1.80) compared to near-perfect training results (R^2^ = 0.93, RPD = 3.89), likely due to excessive reliance on kernel-driven nonlinear mapping (**Figure 7B**). This may be due to the model’s reliance on complex kernel functions that overfit the training data but fail to generalize to unseen spectra with subtle physiological variations.

The PLSR model showed moderate test robustness (R^2^ = 0.75, RPD = 1.99) (**Figure 7A**). However, its linear assumptions constrained its ability to fully capture the complex and nonlinear relationships between spectral features and LNC, especially in the red-edge and near-infrared regions. Notably, the FNN model displayed inverted generalization trends, suggesting suboptimal parameterization of its fuzzy inference system for LNC dynamics (**Figure 7C**).

Overall, the results indicate that SAE-FNN provided the most consistent and accurate predictions of LNC among the evaluated models, while SVM, PLSR, and FNN exhibited varying limitations in modeling high-dimensional spectral data. Similar results were also shown in the five cross validations (**Supplementary Table S3**). It is worth noting that when using PCA for feature extraction instead of CARS, all models showed a significant decrease in predictive performance (**Supplementary Figure S2**), further underscoring the critical importance of effective wavelength selection and the superiority of the CARS method in this framework.

## 4 Discussion

### 4.1 Treatment-specific dynamics of LNC and spectral characteristics

The LNC of tomato plants exhibited pronounced treatment-dependent variations across growth stages, reflecting the critical influence of nitrogen and water management on physiological dynamics. During the seedling stage, T2 achieved the highest LNC, which may result from asynchronous nitrogen release and plant uptake under conventional fertilization and full irrigation, as similarly observed in potato systems (Yan et al., 2012; Yang et al., 2020). In contrast, T5 showed the lowest LNC, consistent with nitrogen limitation under reduced fertilization and water stress (**Figure 2A**). Compared to full irrigation (T3), deficit irrigation (T5) reduced LNC by 6.14%. These results suggest that water availability not only affects nutrient mass flow to roots but also regulates root metabolic activity and nitrogen transporter expression, influencing nitrogen uptake efficiency in early vegetative stages.

During the flowering stage, T3 achieved the highest LNC (**Figure 2B**), which can be attributed to the synergistic effects of optimized nitrogen management and adequate water supply, enhancing nitrogen availability, assimilation, and allocation to photosynthetic tissues (Abdelghany et al., 2023). By the fruit development stage, T2 regained dominance, reflecting the shift in nitrogen remobilization priorities toward fruit development (**Figure 2E**). This aligns with previous studies showing that during rapid fruit growth (35–50 days post-anthesis), tomato plants prioritize 60–70% of nitrogen uptake to fruits, resulting in intense intra-plant competition (Wang et al., 2019). The higher nitrogen supply under full fertilization (CF) supports prolonged xylem-based transport, favoring nitrogen accumulation in the fruits, while optimized fertilization (OF) relies more on phloem-based redistribution (Abdelghany et al., 2023). This period coincides with peak vegetative demand for nitrogen to support leaf expansion and chlorophyll synthesis, and sufficient water ensures hydraulic conductance and nitrogen transport via xylem. As a result, nitrogen uptake and assimilation are maximized, enabling better canopy development and photosynthetic efficiency (Raj et al., 2021). Thus, it is emphasizing the physiological trade-off between vegetative maintenance and reproductive allocation under constrained nitrogen or water conditions.

The temporal dynamics of chlorophyll content, as measured by SPAD values, corroborated the LNC and spectral findings (**Supplementary Figure S1**). The SPAD data revealed a clear, treatment-dependent hierarchy throughout the growth cycle. T2 consistently maintained the highest chlorophyll content, followed by T3, with the water-saving treatments (T4 and T5) showing intermediate values and T1 exhibiting the lowest SPAD readings. This hierarchy aligns perfectly with the gradient of nitrogen availability across the treatments, highlighting the intrinsic link between nitrogen nutrition and chlorophyll synthesis.

Spectral reflectance patterns revealed clear nitrogen- and stage- specific variations (**Figure 3**). At the seedling stage, minimal spectral divergence were observed across treatments, except for T2, which exhibited elevated reflectance at 700 nm. During the flowering stage, T3 exhibited maximal reflectance within the 600–700 nm chlorophyll absorption region, corresponding with its peak LNC (**Figure 3B**). In contrast, T4 and T5 demonstrated reduced reflectance above 690 nm, likely due to lower chlorophyll and water content under nitrogen stress. During the fruit setting stage, attenuated reflectance across all treatments mirrored nitrogen remobilization for fruits development, consistent with observed in oilseed rape (Yu et al., 2018). These spectral shifts align with known nitrogen–chlorophyll–water interactions, where nitrogen deficiency disrupts pigment synthesis and hydration, altering light absorption in visible and near-infrared regions.

Through a comprehensive analysis of the dynamics of leaf nitrogen content in tomato plants, it is evident that the timing and dosage of nitrogen management, along with water supply, play a crucial role in modulating nitrogen content. These changes are significantly reflected in spectral reflectance patterns, providing a valuable tool for monitoring plant nitrogen status in real-time. The observed spectral shifts, particularly in the chlorophyll absorption region, demonstrate the complex interplay between nitrogen availability, leaf pigment synthesis, and water status (Huang et al., 2024; Rubo and Zinkernagel, 2022).

### 4.2 Spectral preprocessing effect and feature band screening

#### 4.2.1 Spectral preprocessing and its effects

To reduce noise and improve spectral fidelity, SG smoothing and SNV normalization were applied (**Figure 4**). SG smoothing effectively suppressed high-frequency noise while preserving critical nitrogen-related spectral features, particularly around 700 nm (**Figure 4B**). SNV normalization minimized baseline shifts caused by surface heterogeneity, centering reflectance values around zero and enhancing sensitivity in the red-edge region (730–780 nm), which is closely associated with chlorophyll content and nitrogen status (**Figure 4D**). These preprocessing steps significantly improved the SNR (**Figure 5A**), thereby clarifying the nonlinear relationships between spectral features and LNC (Liu et al., 2019).

#### 4.2.2 Mechanism and comparison of feature band selection

CARS significantly outperformed PCA (**Table 4**). Unlike the unsupervised variance maximization of PCA (Elhaik, 2022), CARS incorporates the response variable into its iterative Monte Carlo sampling and exponential decay mechanisms. This enables the selection of key wavelengths with high information content and low redundancy (Zhang et al., 2020). The built-in competitive screening mechanism further regularizes high-dimensional, small-sample datasets, reducing overfitting risk. Therefore, CARS achieved outstanding predictive performance using only eight feature wavelengths (e.g. 725 nm and 760–765 nm; see **Figure 5B**). which are physiologically linked to chlorophyll synthesis and mesophyll structural variation (Ihuoma and Madramootoo, 2020; Zhu et al., 2020).

The selected bands are supported by biophysical and chemical principles. Around 725 nm, absorption arises from C–H harmonic vibrations and N–H bond combinations, reflecting amino group density in proteins and chlorophyll, as well as nitrogen assimilation-related carbon skeleton synthesis (Wang et al., 2021; Rasooli Sharabiani et al., 2023). Furthermore, the red edge region at 730–780 nm is directly influenced by the electronic transitions (Qy band) of chlorophyll molecules and affected by internal scattering effects within the mesophyll. Under nitrogen-sufficient conditions, increased chlorophyll synthesis enhances absorption at 680 nm, and shift the red edge to longer wavelengths (Padilla et al., 2015; Tian et al., 2011). Consequently, these mechanisms explain why the red-edge region serves as a reliable proxy for nitrogen status.

#### 4.2.3 The physiological significance and universality of spectral characteristics

This study identified the characteristic wavelength band (690–750 nm), which maps three levels of physiological response mechanisms: (1) chlorophyll-protein coupling (Qy transition of Chl a) at 690 nm, directly regulated by nitrogen-dependent apolipoprotein synthesis (Wang et al., 2017). (2) The red-edge displacement beyond 730 nm, driven by nitrogen induced changes in mesophyll thickness and cellular architecture (Padilla et al., 2015). And (3) a reactivation signal near 765 nm, reflecting the shift in pigment absorption from chlorophyll to carotenoids and proteins during senescence (Kobayashi et al., 1996).

Notably, these spectral-anatomical correlations are species-specific. For example, in maize with Kranz anatomy, the red edge position is shifted 15–20 nm higher than in C3 crops such as tomatoes (Shu et al., 2023), correlating more strongly with mesophyll tissue thickening (Raj et al., 2021), whereas in sugar beet, shifts are related to palisade cell elongation and chloroplast accumulation (He et al., 2020). These inter-species differences suggest that while red-edge features serve as universal proxies for nitrogen status, crop-specific calibration is essential for accurate diagnostics. Even within the C3 crop genus Brassica, the VIP method identified 445 nm (the Chlb Soret band) as a key wavelength reflecting differences in pigment allocation strategies among genotypes (Li et al., 2018). Therefore, while the red-edge region (680–780 nm) serves as a universal indicator of nitrogen status, accurate diagnostics require crop-specific calibration (Zhu et al., 2014). Integrating spectral responses with underlying biochemical and anatomical mechanisms will enhance the ecological validity and universality of hyperspectral monitoring tools.

### 4.3 Spectral-based inversion model for nitrogen content in tomato leaves

Among the evaluated models, the SAE-FNN model demonstrated superior performance in predicting tomato leaf nitrogen content. This improvement is attributed to its dual capacity to hierarchically extract deep spectral features and model nonlinear nitrogen-reflectance relationships. The SAE isolates key nitrogen-sensitive spectral signatures (700–780 nm) by suppressing confounding noise through unsupervised pre-training (Liang et al., 2018). Subsequently, the FNN fine-tunes these features via backpropagation, explicitly capturing nonlinear interactions between nitrogen content and red-edge displacement dynamics (>730 nm), which are known to reflect chlorophyll and nitrogen variations. This hybrid model achieved the highest validation performance (R^2^ = 0.74, RPD = 1.96), outperforming a standalone FNN (R^2^ = 0.67) (**Figure 7**), and confirming that deeper network architectures are essential for disentangling complex biochemical- spectral associations (Yu et al., 2018). The SAE-FNN’s success thus stems from its theoretical foundation in deep representation learning, effectively tackling the high dimensionality and inherent nonlinearities of the problem.

The superiority of SAE-FNN is consistent with findings in other crops. In maize, Goel et al. (2003) reported that deep learning models, artificial neural networks (ANNs) outperformed decision tree in predicting canopy nitrogen content using hyperspectral imagery. Similarly, Zhang et al. (2023a) demonstrated enhanced prediction accuracy for chlorophyll content in Chinese cabbage vegetables using deep learning architectures self-adjusted One-Dimensional Convolutional Neural Network (SA-1DCNN), and Yi et al. (2007) reported robust nitrogen estimation in rice using artificial neural networks (ANN). These cross-crop results reinforce the general advantage of deep learning models in handling high-dimensional, nonlinear hyperspectral data for nitrogen monitoring.

Furthermore, the SVM exhibited significant overfitting in this study, likely due to the sensitivity to kernel-induced complexity in high-dimensional spaces (**Figure 7B**). SVM operates by constructing a maximum-margin hyperplane in a transformed feature space via a kernel function. However, in hyperspectral data with extreme dimensionality, such transformations can inadvertently amplify noise and lead to overfitting (Asefa et al., 2005). Previous studies have recommended stronger regularization (e.g., C ≥ 10^4^) to mitigate this risk in similar applications (Li et al., 2016; Yang et al., 2018). Although SVMs are theoretically well-suited for moderate-dimensional regression tasks, they may lack scalability and robustness for nitrogen estimation tasks in greenhouse environments where multiple spectral, environmental, and developmental variables interact. Without extensive and computationally intensive parameter tuning, SVMs may therefore be less appropriate for modeling complex hyperspectral relationships (Cawley and Talbot, 2010).

While SAE-FNN showed clear advantages in accuracy and generalization, its computational demands and reliance on large training datasets pose challenges for real-time field deployment (**Figure 7D**). This constrains its immediate deployment of SAE-FNN models in resource-limited settings or for real-time decision-making. To address this, future research should explore lightweight variants (e.g., pruning-based FNNs or knowledge distillation), or apply transfer learning with pre-trained spectral encoders to reduce training time while preserving accuracy. Yu et al. (2018) have demonstrated the potential of such approaches in improving model transferability across crop types and environmental conditions.

Overall, the integration of CARS-based feature selection with the SAE-FNN model provides a biologically interpretable and technically robust framework for non-destructive nitrogen diagnosis in tomato. CARS provides a biologically grounded starting point by focusing on wavelengths known for nitrogen sensitivity, enhancing interpretability (Li et al., 2009). By targeting red-edge wavelengths closely associated with nitrogen-induced physiological changes, this approach ensures high prediction accuracy while maintaining model generalizability across growth stages. Its practical value lies in enabling real-time monitoring of plant nitrogen status, which is critical for precision nutrient regulation in greenhouse environments. Similar strategies have shown promise in maize and rice systems, where red-edge features were successfully applied for canopy-level nitrogen monitoring using deep models (Song et al., 2019; Yi et al., 2007).

In practical applications, this hyperspectral-deep learning framework could be embedded into fertigation decision-support systems or multispectral imaging devices for automated nitrogen scheduling, improving nitrogen use efficiency and crop quality in facility agriculture. The selected key bands (e.g., 725 and 765 nm) can be translated into low-cost sensors integrated on trolleys, UAVs, or fixed greenhouse infrastructure. To further enhance field applicability, future efforts should focus on model lightweighting and transfer learning, enabling broader deployment across cultivars and environments. This research thus lays the foundation for intelligent crop management systems that combine physiological insight with spectral automation.

While this study demonstrates a promising framework, several aspects warrant further development. The generalizability of the models requires validation across diverse cultivars and environments beyond the controlled greenhouse conditions. The sample size, though sufficient for current analysis, could be expanded into larger, multi-season datasets to strengthen robustness. Furthermore, translating leaf-level spectroscopy into practical field applications requires future research into canopy-level remote sensing via UAVs. Finally, developing more interpretable and computationally efficient models will be essential for real-world deployment. Future efforts should integrate real-time sensing into automated management systems to achieve sustainable precision agriculture.

## 5 Conclusions

This study demonstrated the feasibility and robustness of using hyperspectral imaging (HSI) combined with advanced machine learning models for dynamic, non-destructive monitoring of nitrogen content in greenhouse-grown tomato plants under varying water and nitrogen management regimes. LNC exhibited pronounced stage- and treatment-specific dynamics, particularly in the red-edge region (730–780 nm). The integration of CARS for feature selection and a SAE-FNN for modeling significantly improved prediction accuracy and model generalization, outperforming conventional approaches such as PLSR and SVM. The findings clarify the potential of hyperspectral-based monitoring frameworks to support precision nitrogen management in protected vegetable production systems. Future efforts should focus on developing lightweight network architectures and transfer learning strategies to facilitate real-time deployment at scale. Integrating such systems into greenhouse fertigation control platforms may enable intelligent, responsive nutrient regulation, advancing the digital transformation of sustainable vegetable production.

## Data Availability

The raw data supporting the conclusions of this article will be made available by the authors, without undue reservation.
