# Supplementary material for: Visible-near infrared hyperspectral imaging for non-destructive estimation of leaf nitrogen content under water-saving irrigation in protected tomato cultivation
Source: Front Plant Sci. 2025 Oct 1;16:1676457. doi: 10.3389/fpls.2025.1676457 (PMC12521146; doi:10.3389/fpls.2025.1676457)
Supplement: Supplementary file 1 [file DataSheet1.docx]

*Supplementary information*

Visible-near infrared hyperspectral imaging for non-destructive estimation of leaf nitrogen content under water-saving irrigation in protected tomato cultivation

Caixia Hu ^1^, Tingting Zhao ^1^, Yingying Duan ^2^, Yungui Zhang ^2^, Xinxiu Wang ^2^, Jie Li ^1*^, Guilong Zhang ^1*^

^1^ Agro-Environmental Protection Institute, Ministry of Agriculture and Rural Affairs, Tianjin, 300191, China

^2^ Institute of Agricultural Resources and Regional Planning, Chinese Academy of Agricultural Sciences, Beijing, 10081, China

* Corresponding author.

*E-mail addresses:* [lijie@caas.cn](mailto:lijie@caas.cn) (J. Li), [zhangguilong@caas.cn](mailto:zhangguilong@caas.cn) (G. Zhang).

**TABLE S1** Sensitive wavelengths selected by competitive adaptive reweighted sampling (CARS) and the highest weighted wavelengths in the first four principal components (PCs) from principal component analysis (PCA)

| **Methods** | **Selected Components / Wavelengths (nm)** |
| --- | --- |
| CARS | 510, 515, 645, 725, 760, 765, 865, 870. |
| PCA | Components 1: 475, 490, 470, 455, 500; Components 2: 690, 695, 700, 685, 705; Components 3: 740, 730, 735, 745, 725; Components 4: 745, 740, 750, 735, 755. |

**TABLE S2** Optimized hyperparameters and model configurations for the four machine learning approaches

| **Model** | **Parameters** |
| --- | --- |
| PLSR | Copy: True, Max_iter: 500, n_components: 5, Scale: True, Tol: 1e-06. |
| SVM | C: 372.4874, Epsilon: 0.3039, Kernel: rbf, Degree: 3, Gamma: scale, Coef0: 0.0, Tol: 0.001, Shrinking: True, Cache_size: 200, Max_iter: -1. |
| FNN | Input Dimension:121, Hidden Layer 1 Units: 76, Hidden Layer 2 Units: 73, Learning Rate: 0.0047, L2 Regularization: 0.059, Dropout Rate: 0.4141, Optimizer: Adam, Loss Function: Mean Squared Error, Batch Size: 64, Maximum Epochs: 200, Early Stopping Patience: 15. |
| SAE-FNN | Input Dimension: 121, Encoder Dimensions: [197, 49], Learning Rate: 0.0023, L2 Regularization: 0.0190, Dropout Rate: 0.1534, Optimizer: Adam, Loss Function: Mean Squared Error, Batch Size: 64, Autoencoder Epochs: 50, Predictor Epochs: 200, Early Stopping Patience: 15. |

**TABLE S3** Five-fold cross-validation performance metrics of PLSR, SVM, FNN and SAE-FNN models were based on the optimal CARS feature extraction method.

| **Model** | **Fold** | **Train R^2^** | **Train RMSE** | **Train RPD** | **Validation R^2^** | **Validation RMSE** | **Validation RPD** |
| --- | --- | --- | --- | --- | --- | --- | --- |
| PLSR | 1 | 0.72 | 0.13 | 1.89 | 0.48 | 0.19 | 1.38 |
|  | 2 | 0.62 | 0.15 | 1.63 | 0.79 | 0.11 | 2.15 |
|  | 3 | 0.67 | 0.14 | 1.74 | 0.53 | 0.16 | 1.46 |
|  | 4 | 0.66 | 0.15 | 1.72 | 0.69 | 0.15 | 1.80 |
|  | 5 | 0.69 | 0.14 | 1.20 | 0.32 | 0.18 | 1.21 |
|  | Mean | 0.67 | 0.14 | 1.76 | 0.56 | 0.16 | 1.60 |
| SVM | 1 | 0.92 | 0.07 | 3.63 | 0.61 | 0.17 | 1.59 |
|  | 2 | 0.94 | 0.06 | 4.03 | 0.83 | 0.10 | 2.42 |
|  | 3 | 0.93 | 0.07 | 3.74 | 0.70 | 0.13 | 1.83 |
|  | 4 | 0.936 | 0.06 | 3.96 | 0.55 | 0.18 | 1.49 |
|  | 5 | 0.94 | 0.06 | 4.09 | 0.76 | 0.13 | 1.71 |
|  | Mean | 0.93 | 0.07 | 3.89 | 0.69 | 0.14 | 1.81 |
| FNN | 1 | 0.74 | 0.13 | 1.98 | 0.69 | 0.13 | 1.80 |
|  | 2 | 0.77 | 0.12 | 2.10 | 0.81 | 0.10 | 2.27 |
|  | 3 | 0.73 | 0.13 | 1.93 | 0.55 | 0.17 | 1.50 |
|  | 4 | 0.73 | 0.14 | 1.92 | 0.56 | 0.14 | 1.51 |
|  | 5 | 0.73 | 0.12 | 1.92 | 0.63 | 0.19 | 1.64 |
|  | Mean | 0.74 | 0.13 | 1.96 | 0.63 | 0.19 | 1.64 |
| SAE-FNN | 1 | 0.90 | 0.07 | 2.75 | 0.79 | 0.10 | 2.18 |
|  | 2 | 0.89 | 0.08 | 2.63 | 0.76 | 0.11 | 2.02 |
|  | 3 | 0.89 | 0.08 | 2.68 | 0.70 | 0.12 | 1.99 |
|  | 4 | 0.89 | 0.09 | 2.60 | 0.77 | 0.11 | 2.08 |
|  | 5 | 0.88 | 0.08 | 2.66 | 0.68 | 0.11 | 1.83 |
|  | Mean | 0.89 | 0.08 | 2.6 | 0.74 | 0.11 | 2.02 |


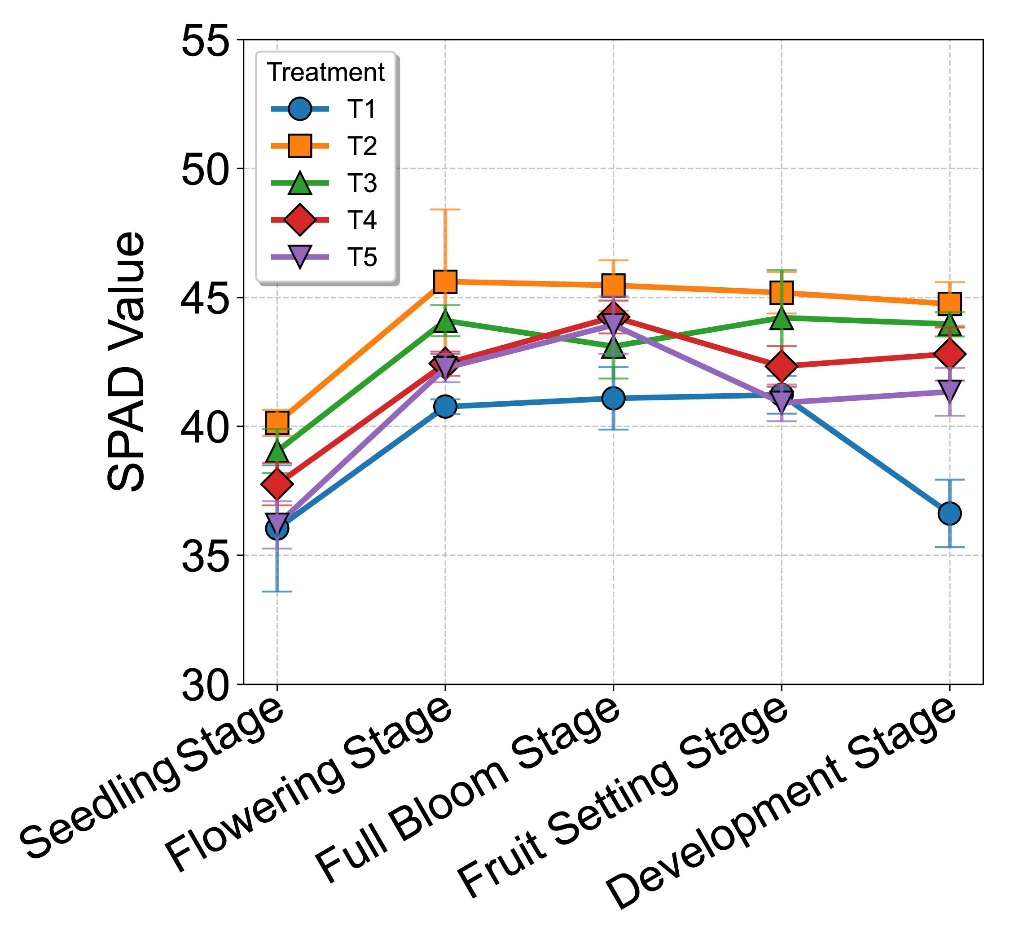


**FIGURE S1** SPAD values across different growth stages and treatments


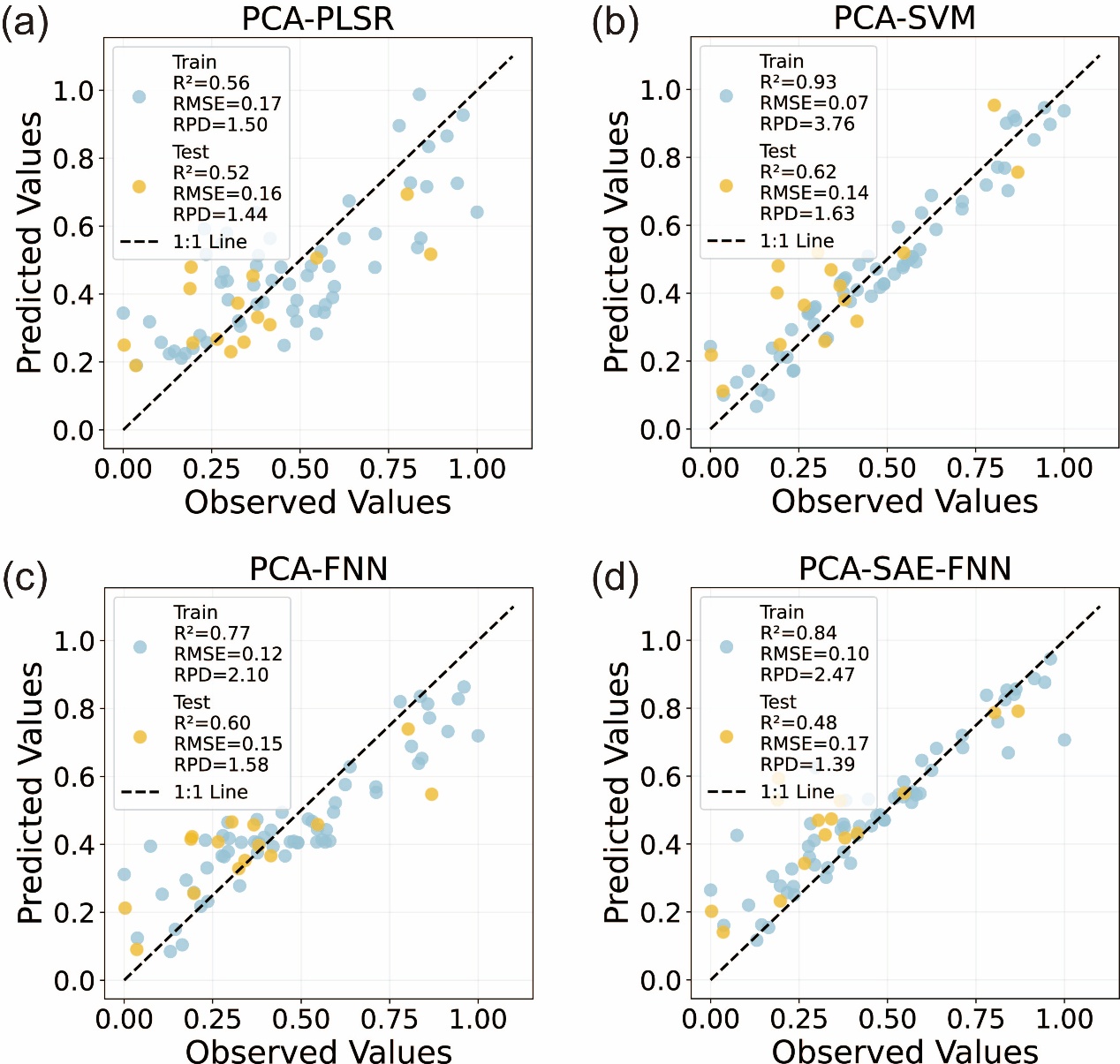


**FIGURE S2** The predictive performance of the four machine learning models (PLSR, SVM, FNN and SAE-FNN) were based on the PCA feature extraction method.
